# Pulse-Modulated Radio-Frequency Alternating-Current-Driven Atmospheric-Pressure Glow Discharge for Continuous-Flow Synthesis of Silver Nanoparticles and Evaluation of Their Cytotoxicity toward Human Melanoma Cells

**DOI:** 10.3390/nano8060398

**Published:** 2018-06-02

**Authors:** Anna Dzimitrowicz, Aleksandra Bielawska-Pohl, George C. diCenzo, Piotr Jamroz, Jan Macioszczyk, Aleksandra Klimczak, Pawel Pohl

**Affiliations:** 1 Department of Analytical Chemistry and Chemical Metallurgy, Faculty of Chemistry, Wroclaw University of Science and Technology, Wybrzeze St. Wyspianskiego 27, 50-370 Wroclaw, Poland; anna.dzimitrowicz@pwr.edu.pl (A.D.); piotr.jamroz@pwr.edu.pl (P.J.); 2 Laboratory of Biology of Stem and Neoplastic Cells, Hirszfeld Institute of Immunology and Experimental Therapy Polish Academy of Science, R. Weigla 12, 53-114 Wroclaw, Poland; aleksandra.bielawska@iitd.pan.wroc.pl (A.B.-P.); klimczak@iitd.pan.wroc.pl (A.K.); 3 Department of Biology, University of Florence, via Madonna del Piano 6, 50017 Sesto Fiorentino, Italy; georgecolin.dicenzo@unifi.it; 4 Faculty of Microsystem Electronics and Photonics, Wroclaw University of Science and Technology, Wybrzeze St. Wyspianskiego 27, 50-370 Wroclaw, Poland; jan.macioszczyk@pwr.edu.pl

**Keywords:** cold atmospheric-pressure plasma, nanostructures, necrosis

## Abstract

An innovative and environmentally friendly method for the synthesis of size-controlled silver nanoparticles (AgNPs) is presented. Pectin-stabilized AgNPs were synthesized in a plasma-reaction system in which pulse-modulated radio-frequency atmospheric-pressure glow discharge (pm-rf-APGD) was operated in contact with a flowing liquid electrode. The use of pm-rf-APGD allows for better control of the size of AgNPs and their stability and monodispersity. AgNPs synthesized under defined operating conditions exhibited average sizes of 41.62 ± 12.08 nm and 10.38 ± 4.56 nm, as determined by dynamic light scattering and transmission electron microscopy (TEM), respectively. Energy-dispersive X-ray spectroscopy (EDS) confirmed that the nanoparticles were composed of metallic Ag. Furthermore, the ξ-potential of the AgNPs was shown to be −43.11 ± 0.96 mV, which will facilitate their application in biological systems. Between 70% and 90% of the cancerous cells of the human melanoma Hs 294T cell line underwent necrosis following treatment with the synthesized AgNPs. Furthermore, optical emission spectrometry (OES) identified reactive species, such as NO, NH, N_2_, O, and H, as pm-rf-APGD produced compounds that may be involved in the reduction of the Ag(I) ions.

## 1. Introduction

Since ancient times, the many special properties of metallic silver have been well known and widely utilized because of its antibacterial [1], conductive [2], and optical [3] applications. Special attention has been given to silver nanoparticles (AgNPs), as their high surface-area-to-volume ratio [4] increases their number of possible applications. Most commonly, AgNPs are utilized in surface-enhanced Raman spectroscopy [5] and metal-enhanced fluorescence [6]. Furthermore, because of the excellent antibacterial activities of AgNPs, they are included in antibacterial clothing [7].

One promising utilization of AgNPs, and nanoparticles in general, is their application in medicine, for example, serving as drug delivery vectors [8,9]. The antiproliferating and antitumor activities of AgNPs have been the focus of much scientific effort, having been reviewed by Banti et al. and Zhang et al. among others [10,11]. In particular, AgNPs have gained interest in the field of nanomedicine because of their unique properties and their therapeutic potential in the treatment of at least some human cancers, with a particular focus on breast cancer [12,13]. Notably, a significant difference in the effects of AgNPs on tumor and non-tumor cell lines has been observed [14], supporting their potential as a cancer therapy agent. The effects of AgNPs on cancer cells are dependent on the concentration [13] and size [15] of the AgNPs. For example, smaller AgNPs at a lower concentration induced apoptosis and necrosis in a human pancreatic ductal adenocarcinoma cell line, while larger AgNPs at higher concentrations induced autophagy in this cell line [15].

The cytotoxic and genotoxic effects of AgNPs against mammalian cells [16,17], as well as their antimicrobial properties [18], has led to increased demand for methods for the reproducible production of AgNPs with defined characteristics, such as size and shape. An intriguing technique for AgNP synthesis is the use of cold atmospheric-pressure plasmas (CAPPs) in direct interaction with AgNO_3_ solutions. The synthesis of AgNPs using CAPPs is a promising alternative to conventional “wet” chemical methods. It allows for the production of biocompatible AgNPs with higher monodispersity [19], without the use of potentially toxic reducing agents [20,21]. It additionally allows for the control of the optical and granulometric properties of the resulting nanomaterial, while minimizing the number of required steps and manipulations.

Among the different CAAPs applied for the production of Ag nanostructures directly in solutions, high-voltage direct-current-driven atmospheric-pressure glow discharge (dc-APGD) is the most widely used; however, the number of studies devoted to this topic is limited [22,23,24,25,26,27,28,29,30,31,32,33]. Considering experimental setups, stable dc-APGDs generated in contact with non-flowing solutions have been sustained with the aid of Ar [23,29,31,32,33] or He [24,26,27,28,30] jets. Gases were introduced through Cu [29] or stainless steel [23,24,25,26,27,28,30,31,32,33] tubes or capillaries attached to the negative polarity outputs of high voltage direct current (HV-dc) power supplies. Solutions were positively biased by immersing graphite rods [24], Pt rods [29], or Pt foils [23,28,30,31,32,33] into them, to which the ground earth outputs of the supplies were attached, or through the use of another gaseous jet [27]. Generally, these systems use a stationary AgNO_3_ solution [20,23,24,25,26,27,30,31,32,33,34], and the reactors usually run for up to 30 min [31,32], resulting in the production of limited amounts of nanomaterials. However, a few continuous-flow systems have also been developed that significantly improve the production rate [19,22].

In the systems described above, the plasma generated in the gaps between gas-supporting capillaries and AgNO_3_ solutions is a rich source of high-energy electrons (e_g_^−^) that bombard the solution surface. After thermalization (energy loss through multibody interactions with water molecules) of the e_g_^−^ from the gas phase, solvated electrons (e_aq_^−^) are formed in the liquid phase. Redox reactions mediated by the e_aq_^−^ and other reactive species, such as hydrogen radicals (H), hydrogen peroxide (H_2_O_2_), singlet oxygen (O), nitrogen oxide (NO), and hydroxyls (OH), result in particle nucleation and the growth of AgNPs in the solution [22,23,24,25,26,27,28,29,30]. This process can lead to electrostatically stabilized AgNPs without the need for additional capping agents [22,30,33]. However, various stabilizers, such as pectin [19], dextran [29], fructose [30,31,32], sucrose [24], polyvinyl alcohol [23], polyacrylic acid [25], and sodium dodecyl sulfate [19,26,27], are often included to prevent undesirable agglomeration and precipitation of the Ag nanostructures.

We are unaware of any studies making use of pulse-modulated radio-frequency alternating-current-driven atmospheric-pressure glow discharge (pm-rf-APGD) for the synthesis of nanomaterials. However, pm-rf-APGD presents many characteristics that make it an appealing alternative to dc-APGD for the synthesis of AgNPs. In pm-rf-APGD, there is a constant switching in the polarity of the electrodes, resulting in alternating injections of electrons (positive charge of the flowing liquid electrode) and positive ions (negative charge of the flowing liquid electrode). Both the electrons and positive ions contribute to the production of reactive oxygen and nitrogen species (RONS) responsible for the reduction of Ag(I) ions in the liquid phase. This characteristic may allow for the generation of AgNPs with improved monodispersity and long-term stability. By alternating between conditions convenient for reduction of the precursor of AgNPs (when the flowing liquid electrode of pm-rf-APGD has positive polarity) and conditions permissive for AgNPs etching due to acidification of the liquid (when the flowing liquid electrode of pm-rf-APGD has negative polarity), pm-rf-APGD may allow for better control of the shape and size of the AgNPs [34]. Moreover, the use of pm-rf-APGD is expected to reduce the power consumption of the AgNPs synthesis procedure relative to the use of dc-APGD; when 30% or 70% duty cycles are used, the plasma is off for 70% or 30% of the time, respectively [34].

Here, the properties of pm-rf-APGD-mediated AgNPs are explored. The effects of five parameters of this novel plasma-reaction system on the wavelength of the maximum (*λ*_max_) of the localized surface plasmon resonance (LSPR) absorption band of the resultant AgNPs were examined using a response surface design. On the basis of the full quadratic response surface regression model, optimal conditions for the production of AgNPs of the largest and smallest sizes were selected, and the model was validated. Next, AgNPs produced under the conditions for the largest size were further characterized with respect to their optical and morphological properties and their cytotoxic activity toward human melanoma Hs 294T cells. Furthermore, the processes in the gas phase of the pm-rf-APGD were examined by optimal emission spectrometry (OES) in order to identify the reactive species possibly responsible for the synthesis of the Ag nanostructures.

## 2. Materials and Methods

### 2.1. Reagents and Solutions

To obtain a 1000 mg·L^−1^ stock solution of Ag(I) ions, 0.3937 g of solid silver nitrate (AgNO_3_; Avantor Performance Materials, Gliwice, Poland) was dissolved in 250 mL of de-ionized water. The prepared stock solution was diluted 5, 2.86, or 2 times with de-ionized water, yielding final Ag(I) ion concentrations of 200, 350, and 500 mg·L^−1^, respectively. Next, pectin (Agdia-Biofords, Evry Cedex, France), which is a biocompatible stabilizer, was added to aliquots of the working solutions to final concentrations of 0%, 0.25%, or 0.50% (*m*/*v*). This non-toxic biopolymer was included with the aim to stabilize the synthesized Ag nanostructures as well as to prevent their aggregation and sedimentation. All of the reagents were of analytical grade or higher purity. De-ionized water was used in all experiments.

### 2.2. Plasma-Reaction System for the Continuous Synthesis of AgNPs

**Figure 1:**
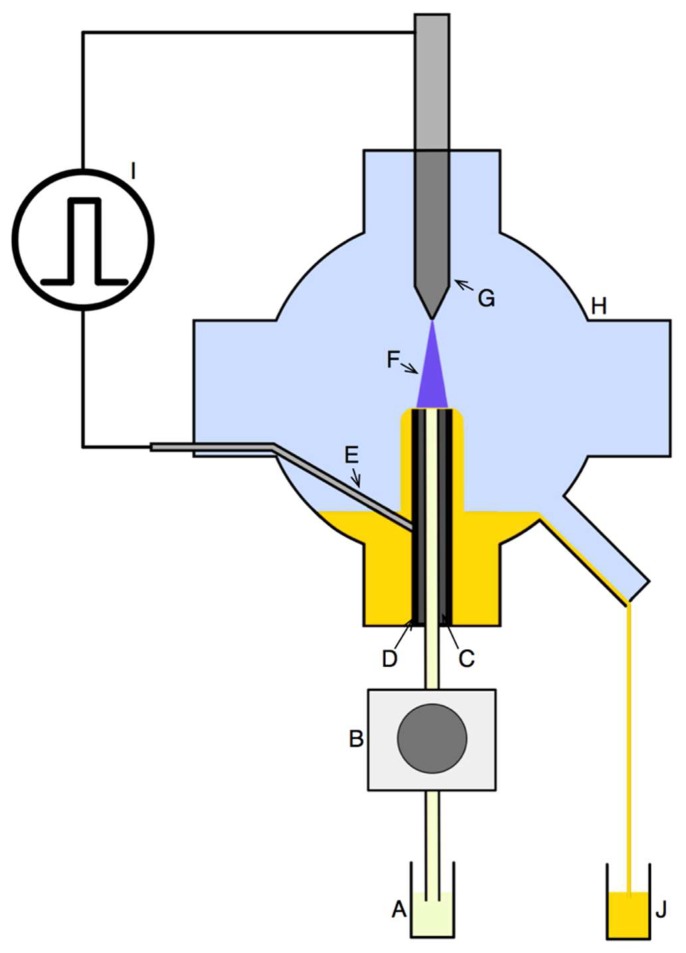
The scheme of the pulse-modulated radio-frequency atmospheric-pressure glow discharge (pm-rf-APGD)-based plasma-reaction system for the continuous production of silver nanoparticles (AgNPs). (A) Solution containing the AgNP precursor with or without pectin; (B) peristaltic pump; (C) quartz capillary; (D) graphite tube; (E) platinum wire; (F) pm-rf-APGD; (G) pin-type electrode; (H) quartz chamber; (I) radio-frequency (rf) voltage; (J) solution after pm-rf-APGD treatment containing AgNPs.

Ag nanostructures were continuously produced in an innovative plasma-reaction system (Figure 1), in which APGD was generated between the surface of the flowing liquid electrode and a pin-type tungsten electrode (outer diameter of 4.00 mm). The APGD was generated using the rf (50 kHz) voltage wave modulated at frequencies within 500–1500 Hz with duty cycles of 30–70%. The distance between the surface of the flowing liquid electrode and the pin-type tungsten electrode was 4.00 mm. The rf voltage waveform was generated using a rf generator (Dora Electronics Equipment, Wroclaw, Poland). To charge the working solutions, a platinum wire was connected to the quartz-graphite capillary. Current and voltage measurements were made using a Rogowski coil and a voltage probe. The measured root-mean-square current was ~15 mA, while the root-mean-square voltage was 5 kV. The working solutions of the flowing liquid electrode were introduced to the developed pm-rf-APGD plasma-reaction system by a four-channel peristaltic pump (Masterflex L\S, Cole-Parme, Vernon Hill, IL, USA), at flow rates of 3.0–6.0 mL·min^−1^ *via* a quartz-graphite capillary (outer diameter of 6.00 mm). In the flowing liquid electrode working solutions, the concentration of Ag(I) ions was between 200 and 500 mg·L^−1^, and the pectin concentration was within 0.00–0.50% (*m*/*v*). Flowing liquid electrode solutions containing Ag(I) ions of defined concentrations (with or without pectin) were continuously introduced to the plasma-reaction system and treated by pm-rf-APGD. Plasma-treated solutions containing AgNPs were collected and subjected to analysis by UV/Vis absorption spectrophotometry to acquire the *λ*_max_ value of the LSPR absorption band, characteristically in the range of 400–750 nm for spherical AgNPs [19,35].

### 2.3. Response Surface Model and Optimization of AgNP Synthesis

To examine the effects of the operating parameters of the pm-rf-APGD plasma-reaction system on the location of *λ*_max_ of the LSPR absorption band of the produced AgNPs, as well as to optimize this system so as to fabricate Ag nanostructures of a given size, the response surface methodology (RSM) was used to plan the experimental treatments and analyze the response surface. In this case, the Box–Behnken experimental design (BBD) was used, and the following five operating parameters were considered: the flow rate of the flowing liquid electrode solution (A) (mL·min^−1^), the precursor concentration in the flowing liquid electrode solution (B) (mg·L^−1^), the stabilizer concentration (C) (% (*m*/*v*)), the frequency of pulse modulation of the rf current (D) (Hz), and the duty cycle (E) (%). The response surface design included 43 randomized experimental treatments at three different levels (−1, 0, and +1) of the operating parameters, counting three center points. The levels of parameters were arbitrarily selected and limited to the range in which stable operation of the pm-rf-APGD plasma-reaction system was obtained, that is, 3.0–6.0 mL·min^−1^ (A), 200–500 mg·L^−1^ of Ag(I) ions as AgNO_3_ (B), 0.00–0.50% (*m*/*v*) of pectin (C), 500–1500 Hz (D), and 30–70% (E). All experimental treatments were executed in one block within the response surface design matrix (with actual and coded values of the operating parameters) that is given in Table 1.

To provide a good assessment of the curvature in the system, the acquired *λ*_max_ values were modeled using a complete quadratic function, including the main and quadratic effects, and the two-way interactions. To lower the dimensionality of the model for easier interpretation, insignificant terms were eliminated using a forward-selection-of-terms algorithm. The suitability of the model was evaluated using an analysis of variance (ANOVA) test, on the basis of the *p*-values for each of the terms in the model as well as the values of the residual standard deviation (*S*) and the coefficient of determination (*R*^2^). The model was also tested for lack-of-fit, which provided evidence of the adequacy and efficacy of the full quadratic regression model for approximation of the response surface. In such a case, the respective *p*-value for the lack-of-fit test should ideally be high. Finally, the quality of the model and the reliability of its assumptions were checked by examining the residuals. To do this, scatter plots of the frequency distribution of the standardized residuals, as well as the standardized residuals *versus* fitted values, were used for identifying outliers and/or non-contact variance. This provided a good way to look for severe non-normality or heteroscedasticity (unequal variation) in residuals in the model to ensure informed decisions about acceptance or rejection of the model. The response surface design was planned and analyzed using the Minitab 17 statistical software package (Minitab Ltd., Coventry, UK) for Windows 7 (32 bit).

### 2.4. Characterization of the AgNPs Synthesized under Defined Operating Conditions

The optical properties of the Ag nanostructures were assessed using UV/Vis absorption spectrophotometry. The UV/Vis absorption spectra were acquired in the range from 260 to 1100 nm with a step of 0.1 nm, using a Specord 210 Plus (Analityk Jena, Jena, Germany) double-beam spectrophotometer. The UV/Vis absorption spectra were registered 1440 min after the pm-rf-APGD treatment. De-ionized water was used to zero the instrument. As the recorded UV/Vis absorption spectra were composed of more than a single band, the spectra were deconvoluted to resolve *λ*_max_ of the LSPR absorption band. The acquired UV/Vis absorption spectra were resolved using OriginPro 8 software (OriginLab Corp., Northampton, MA, USA) and were fitted by Gaussian functions, as was done by Calabrese et al. [36].

The size distribution by number and the polydispersity index of the synthesized Ag nanostructures (in reference to the mean hydrodynamic diameter) were estimated using a ZetaSizer Nano-ZS instrument (Malvern Instrument, Malvern, UK) with a detection angle of 173°. The measurements were performed at 25 °C in optically homogenous polystyrene cuvettes. To reveal the surface charge of the AgNPs, the *ξ*-potential was measured with this instrument as well. To assess the results for the particle size distribution, polydispersity index, and *ξ*-potential, which represent the average of three measurements, Malvern Dispersion Technology Software (version 7.11) for ZetaSizer was applied.

Next, the size and shape distributions, as well as the elemental composition and crystallographic structure, of the AgNPs produced under the optimal operating conditions were estimated *via* Tecnai G^2^ 20 X-TWIN transmission electron microscopy (TEM) (FEI Co., Hillsboro, OR, USA) supported by energy-dispersive X-ray spectroscopy (EDS)(FEI Co., Hillsboro, OR, USA) and selected-area electron diffraction (SAED; AztecEnergy, Oxford Instrument, Abingdon, UK). One drop of proper solution was put onto a Cu grid (CF 400-Cu-UL, Electron Microscopy Sciences, Hatfield, PA, USA) and evaporated to dry under infrared (IR) irradiation (95E, Philips Lighting, Pila, Poland). The size and shape distributions were assessed on the basis of the diameter of 60 single nanoparticles using FEI software (version 3.2, SP6 build 421, FEI Co., Hillsboro, OR, USA).

### 2.5. Optical Emission Spectrometry

To identify reactive species generated in the gas phase of the pm-rf-APGD, OES measurements were performed. A Shamrock SR-500i (Andor, Belfast, UK) spectrometer with a Newton DU-920P-OE CCD camera (Andor, Belfast, UK) was used to resolve the radiation emitted by the pm-rf-APGD collimated onto the entrance slit (10 μm).

### 2.6. Purification of AgNPs

Dialysis was applied in order to purify the AgNPs from the pm-rf-APGD-treated reaction mixture, which also contained unreacted Ag(I) ions. The plasma-treated solution was transferred into a dialysis tube (molecular weight cut-off = 14,000 Da; Sigma-Aldrich, Poznan, Poland) and placed into 500 mL of de-ionized water. The dialysis was conducted for 24 h with magnetic stirring (WIGO, Pruszkow, Poland), as was done previously [19].

### 2.7. Determining the Efficiency of AgNP Synthesis

The yield of AgNPs following the pm-rf-APGD treatment was measured before and after dialysis. The yield was estimated by flame atomic absorption spectrometry (FAAS) using a PerkinElmer 1100 B (Waltham, MA, USA), which is a single-beam flame atomic absorption spectrometer. The FAAS measurements were carried out after the digestion of the AgNPs in a 65% (*m*/*m*) HNO_3_ solution (Avantor Performance Materials, Gliwice, Poland) at 100 °C for 30 min.

### 2.8. Cell Culture Conditions

Hs 294T melanoma cells are of a continuous cell line established from metastatic melanoma isolated from a human lymph node. These cells were obtained from the American Type Culture Collection (ATCC HTB-140). The cell line was cultured as monolayers in high-glucose Dulbecco’s modified eagle medium (DMEM) culture medium supplemented with GlutaMAX-I, 10% heat-inactivated fetal bovine serum (BioMin Biotechnologia, Getzersdorf, Austria), 100 μg·mL^−1^ penicillin, and 100 μg·mL^−1^ streptomycin and was maintained in a cell culture incubator at 37 °C in 5% CO_2_.

### 2.9. Experimental Groups and Exposure Conditions

In order to evaluate the effect of AgNPs on necrosis of the Hs 294T cell line, cells were treated with different concentrations of the reaction mixture compounds (for groups 2 and 3) or with the purified and non-purified Ag nanostructures (1, 5, 10, 50, and 100 μg·mL^−1^) for 24 h. As a control, culture medium was used for 24 h (group 1). The details of the experimental protocol are given in Table 2.

### 2.10. Necrotic Assay

Necrosis was measured by flow cytometry to assess the number of human cells incorporating propidium iodide compared to control cells treated only with culture medium alone. Briefly, cells were cultured in medium containing fetal calf serum in 48-well plates and used in the experiments after reaching 80–90% confluence. A defined concentration of AgNPs was added to each well, and the cells were incubated for a further 24 h. A propidium iodide solution (1 μg·mL^−1^) was then added to each sample, and the dead cells were detected using flow cytometry in a FL3 mode, i.e., red channel, λ_em_ = 620 nm. Data were analyzed using a FACSCalibur flow cytometer (Becton Dickinson, Franklin Lakes, NJ, USA). The percentage of necrotic target cells was calculated using the Flowing Software 2 program. All values are presented as the mean ± standard error of the mean of three independent experiments, each consisting of technical triplicates. The results were analyzed through Student’s *t*-tests using GraphPad Prism 5 software. The *p*-values for all investigated groups were calculated compared to the control group 1 (cells treated with medium alone).

## 3. Results and Discussion

### 3.1. Response Surface Regression Model

Nanostructures of different metals and sizes are able to absorb and reflect light of unique wavelengths, resulting in a LSPR absorption band centered around distinct wavelengths known as *λ*_max_ [19]. In the case of spherical AgNPs, the LSPR absorption band typically occurs within the 400 to 750 nm range [14,19], with larger-sized AgNPs resulting in a red shift in the position of the *λ*_max_ values. Therefore, to evaluate the effects of the operating parameters on the size of the synthesized AgNPs, 43 independent runs of AgNPs synthesis were performed with varying experimental conditions, and the position of *λ*_max_ of the LSPR absorption band for each sample was recorded (Table 1).

To evaluate the quality of the data, two scatter plots were prepared: (i) the range of the values of *λ*_max_ measured under a given condition *versus* the mean of the *λ*_max_ values, and (ii) the mean value of the *λ*_max_ values *versus* the randomized run order. It was visually noted that the variability in the mean response between experimental treatments of the BBD was higher than the variability in the response within each treatment (for repeated measurements). Neither correlations nor trends in the scatter plots were observed. Thus, it was concluded that the variability in the results was associated with changes in the experimental conditions and that there was no need to stabilize the variance in the response through mathematical transformation [37,38]. To obtain the response surface, the values of *λ*_max_ for the LSPR absorption band of the 43 AgNP solutions were approximated with a full quadratic polynomial model. The response surface regression model, developed with the aid of the forward-selection-of-terms algorithm, for the *λ*_max_ values over the studied range of operating parameters was as follows (given in uncoded units): *λ*_max_ = 341.8 − 2.34*A* + 3.37 × 10^−2^*B* + 4.27 × 10^−2^*D* + 1.54*E* − 7.16 × 10^−3^*E*^2^ − 7.87 × 10^−4^*DE*. The accuracy of this regression model was tested by ANOVA and the lack-of-fit test. The results of these analyses at α = 0.25 are given in Table 3.

**Figure 2:**
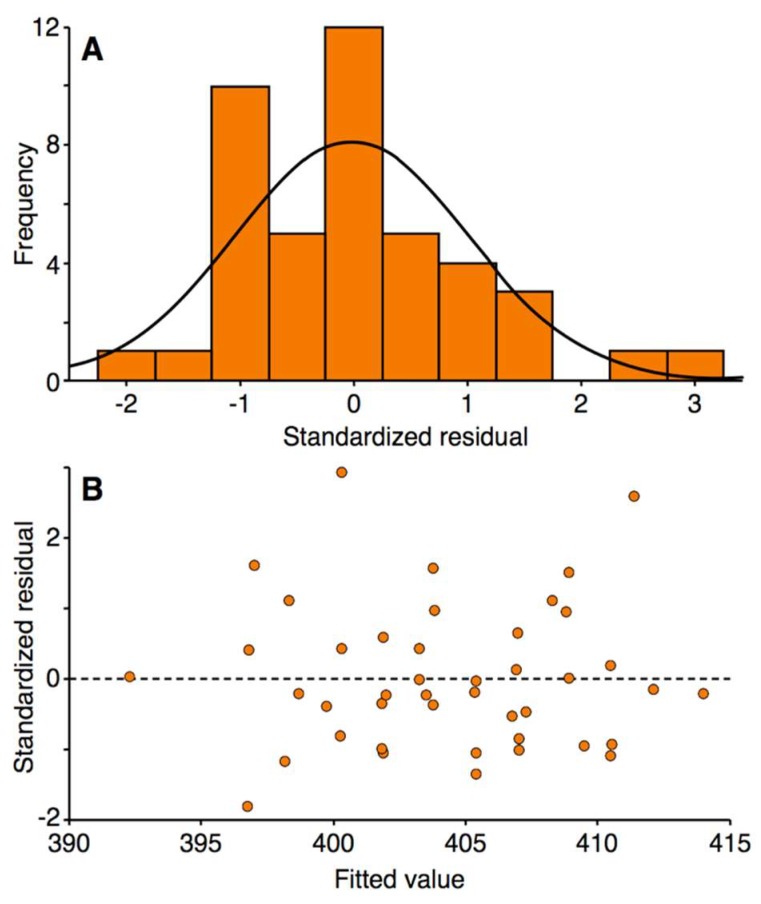
Frequency distribution graph of the standardized residuals (**A**) and a scatter plot of the standardized residuals *versus* the fitted values (**B**).

By using an α value of 0.25, it was possible to learn more about the effects of each of the entered factors on the response and on the terms already in the model [39,40]. The terms A (*p* = 0.074), B (*p* = 0.012), E^2^ (*p* = 0.241) and DE (*p* = 0.046) were statistically significant in the model. Considering the hierarchy of the terms, the statistically insignificant terms D and E (*p* > 0.25) were also included in the model. The concentration of pectin in the flowing liquid electrode solution, that is, term C, appeared to have no statistically significant effect on the position of the *λ*_max_ value of the LSPR absorption band of the synthesized AgNPs. The *R*^2^ value was 32.1%, which was relatively low but acceptable, as it is a measure of how close the data fit the model and not a measure of the adequacy of the regression model. The *p*-value for the lack-of-fit test was higher than 0.25 (*p* = 0.354) and was therefore statistically insignificant. The *S*-value was also relatively low, at 7.61. Therefore, on the basis of the mentioned statistics summarizing the regression model, it was concluded that there was no reason to reject the model nor evidence that it did not fit the data. To finally check the goodness-of-fit of the regression model, the residuals were examined by plotting the following: (i) the frequency distribution of the standardized residuals (Figure 2A), and (ii) the standardized residuals *versus* the fitted values (Figure 2B). The distribution of the standardized residuals largely resembled a normal distribution with a mean of 0.015 and a standard deviation of 1.032 (*n* = 43). A random pattern of the residuals on both sides of zero was observed in the scatter plot of the standardized residuals *versus* the fitted values, indicating that a correct polynomial function was used to model the response surface of the system. Except for two outliers (points 25 and 29), no unusual structures or patterns were observed in this scatter plot. These observations confirmed the correctness of the model and the goodness-of-fit of the empirical data with those established by the regression.

### 3.2. The Effects of Operating Parameters

**Figure 3:**
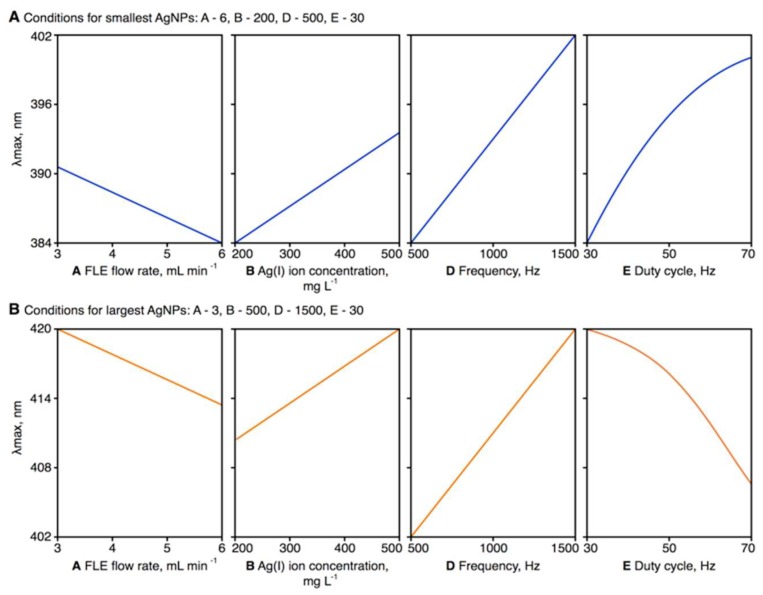
Graphical representation of the effects of the statistically significant parameters included in the response surface regression model on the wavelength at maximum (*λ*_max_) of the localized surface plasmon resonance (LSPR) absorption band of the synthesized silver nanoparticles (AgNPs). Graphs demonstrate the effects of modifying variables when the operating conditions were otherwise optimal for the production of spherical AgNPs with the lowest (**A**) and highest (**B**) *λ*_max_. A: the flow rate of the flowing liquid electrode (FLE) solution (mL·min^−1^); B: the precursor concentration in the FLE solution (mg·L^−1^); D: the frequency of pulse modulation of radio-frequency (rf) current (Hz); E: the duty cycle (%).

The effects of the operating parameters included in the response surface regression model (i.e., A: the flow rate of the flowing liquid electrode solution; B: the precursor concentration in the flowing liquid electrode solution; D: the frequency of pulse modulation of the rf current; and E: the duty cycle) on the position of *λ*_max_ for the LSPR absorption band of the synthesized AgNPs is shown in Figure 3. There was an inversely linear relationship between the flow rate of the flowing liquid electrode solution and the position of the *λ*_max_ values; that is, a higher flow rate was associated with smaller AgNPs. As the flow rate increased, each milliliter of solution was exposed to the pm-rf-APGD for less time. Consequently, a smaller number of solvated electrons (e_aq_^−^) was likely available for reduction of the Ag(I) ions in the plasma-treated solution; that is, Ag^+^ + e_aq_^−^ = Ag^0^ [22,23,24,25,26,34]. Considering the effect of the precursor concentration in the flowing liquid electrode solution, the lowest concentration of Ag(I) ions seemed to be preferable for obtaining small-sized AgNPs, that is, those with the lowest *λ*_max_ value of their LSPR absorption band. This could be explained by the Finke–Watzky two-step mechanism of nucleation and growth of AgNPs [41]. Accordingly, at higher concentrations of Ag(I) ions, faster growth and further aggregation of AgNPs can take place as a result of the reduction of these ions on the surface of the AgNPs. This would make existing AgNPs larger rather then produce new particles in the solution. In addition, at higher Ag(I) concentrations, the ionic strength of the solution could destabilize the synthesized AgNPs [41]. Finally, increasing either the frequency of pulse modulation of the rf current or its duty cycle led to a gradual increase in the position of the *λ*_max_ value of the LSPR absorption band. Hence, AgNPs of a greater particle size would be produced under these conditions. Both of these parameters directly affected the width of the pulses modulating the rf current in the on-and-off cycles and the duration of the interchanges of polarity set to the flowing liquid electrode solution. At the lowest settings of these parameters (i.e., D: 500 Hz; E: 30%), the pulse width at a given modulation frequency will be the shortest, while the time spent in the off state of the cycle will be the longest. Considering that the solution was continuously replenished in the flow-through system used, the above-mentioned conditions would have been responsible for the lowest production yield and the smallest size of the AgNPs. Particularly, the negative polarity of the flowing liquid electrode solution and the bombardments of the solution surface with positive ions, for example, H_2_O^+^_(g)_, could provide convenient conditions for the etching of AgNPs. This is because H_2_O molecules are ionized under these conditions (H_2_O^+^_(g)_ + H_2_O = 2H_2_O^+^_(aq)_ + e_aq_^−^), while the resultant H_2_O^+^_(aq)_ ions recombine with water molecules, leading to the formation of H_3_O^+^(aq) ions and the acidification of the solution (H_2_O^+^_(aq)_ + H_2_O = H_3_O^+^_(aq)_ + OH^•^_(aq)_) [37,38]. This would be a process competitive to the formation of AgNPs through the reduction of the Ag(I) ions; however, it appeared that the longest off time in the cycle was convenient for rearrangement of synthesized nanostructures, leading to improved control over their size. The effect of the stabilizer concentration (*C*) was established to be statistically insignificant. This could be due to electrostatic stabilization of AgNPs, that is, negative charging of the surface of the AgNPs by electrons “injected” from the discharge through the plasma–liquid interface. This explanation was also suggested by Patel et al. [42], who reported surfactant-free synthesis of AuNPs facilitated by dc-APGD operated between a He jet (acting as a cathode) and a non-flowing solution of AuCl_4_^−^ ions (acting as an anode).

### 3.3. Validation of the Response Surface Regression Model

**Figure 4:**
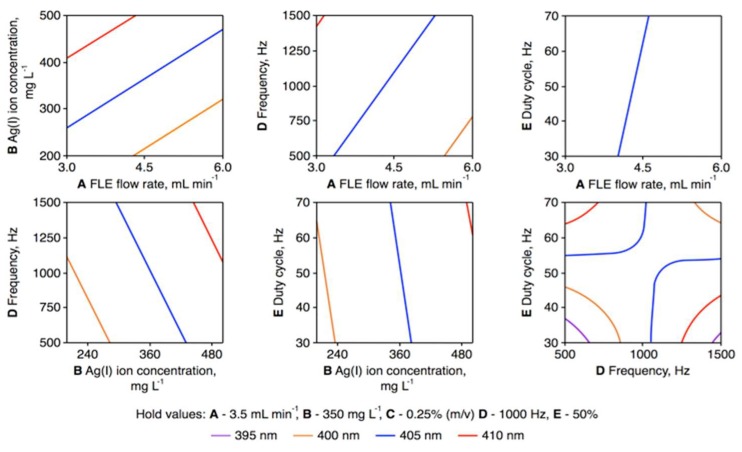
Counter plots of the wavelength at maximum (*λ*_max_) of the localized surface plasmon resonance (LSPR) band of silver nanoparticles (AgNPs) synthesized using the pulse-modulated radio-frequency atmospheric-pressure glow discharge (pm-rf-APGD) system for pairs of statistically significant parameters in the developed response surface regression model (A–B, A–D, A–E, B–D, B–E, and D–E) at defined hold values. A: the flow rate of the flowing liquid electrode (FLE) solution (mL·min^−1^); B: the precursor concentration in the FLE solution (mg·L^−1^); D: the frequency of pulse modulation of rf current (Hz); E: the duty cycle (%).

Graphical illustrations of the response surface regression model for the position of the *λ*_max_ value for the LSPR absorption band of the synthesized AgNPs are given in Figure 4. These are given as contour plots of the *λ*_max_ value for the following pairs of parameters at specific hold values: A–B, A–D, A–E, B–D, B–E, and D–E. To validate the developed regression model, two opposite sets of operating parameters were selected: condition 1, which led to the production of the smallest-size AgNPs (A: 6.0 mL·min^−1^; B: 200 mg·L^−1^; D: 500 Hz; E: 30%), and condition 2, which led to the production of the largest-size AgNPs (A: 3.0 mL·min^−1^; B: 500 mg·L^−1^; D: 1500 Hz; E: 30%). In both cases, pectin was included as a stabilizer in the flowing liquid electrode solutions at a concentration of 0.25%.

**Figure 5:**
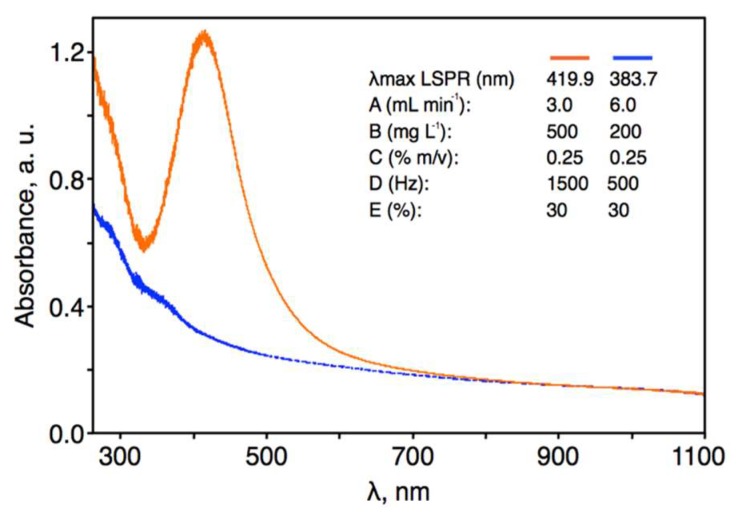
UV/Vis absorption spectra of silver nanoparticles (AgNPs) synthesized with the aid of the pulse-modulated radio-frequency atmospheric-pressure glow discharge (pm-rf-APGD) plasma-reaction under experimental conditions providing the smallest (condition 1) and the largest (condition 2) AgNPs according to the developed response surface regression model.

According to the established regression model, the predicted value of *λ*_max_ of the LSPR absorption band for condition 1 was 383.7 nm, with a standard error of fit of 5.7 nm and a 95% confidence interval of 372.2–395.3 nm. The pm-rf-APGD plasma-reaction system was run with the parameter settings of condition 1, the plasma-treated solutions were collected, and the positions of the *λ*_max_ value of the LSPR absorption bands were determined (Figure 5). The mean value (*n* = 4) of the *λ*_max_ values of the LSPR band was 374.6 ± 3.4 nm with a 95% confidence interval of 371.1–378.0 nm. This corresponded well with the value predicted from the response surface regression model; the absolute error was 9.1 nm, which was lower than two standard errors of fit. Considering condition 2, the fitted value of *λ*_max_ of the LSPR absorption band was 419.9 nm, with a standard error of fit of 5.7 nm and a 95% confidence interval of 408.3–431.5 nm. The plasma-reaction system was run with the parameters of condition 2, and the positions of the *λ*_max_ values for the LSPR absorption band of the synthesized AgNPs were determined (Figure 5). The mean (*n* = 4) *λ*_max_ of the LSPR band was 415.8 ± 5.4 nm with a 95% confidence interval of 410.4–421.2 nm. The absolute error between the predicted and empirical values of *λ*_max_ was just 4.1 nm, which was lower than a single standard error of fit. These tests thus confirmed the usefulness of the developed response surface regression model for the control of the continuous-flow, one-step production process of spherical AgNPs of a given size.

As was done in previous work [42,43,44], the absorbance at *λ*_max_ of the LSPR bands and their full width at half maximum (FWHM) values were recorded as measures of production yield and particle size distribution, respectively, of the AgNPs synthesized using conditions 1 and 2. Under condition 1, which was optimal for obtaining the smallest nanostructures, the absorbance was relatively low (0.21 ± 0.02), which was suggestive of a rather low production yield. The FWHM of the LSPR absorption band of these AgNPs was 106.8 ± 8.0 nm. Additionally, these AgNPs were stable for only 1 week, after which the color intensity of the solutions gradually decreased until they were colorless after 2 weeks. In the case of condition 2, which was optimal for the largest nanostructures, the measured absorbance was fairly high (1.16 ± 0.18), which was indicative of a much higher production yield than that using condition 1. Interestingly, the FWHM of the LSPR absorption bands for AgNPs produced under condition 2 (103.4 ± 6.2 nm) was very similar to that observed for AgNPs produced with condition 1. This suggested that a similar particle size distribution was achieved under both conditions, likely as a result of the electrostatic stabilization provided by the discharge as well as the presence of pectin. The solutions of AgNPs produced using condition 2 remained stable even 6 weeks following plasma treatment, as the shape and absorbance of the LSPR absorption band did not change within this time period. Considering the production yields and the stabilities of the AgNPs synthesized under conditions 1 and 2, only the AgNPs produced under condition 2, optimal for production of the largest nanostructures, were further characterized. According to Mie’s scattering theory [45], a single intense LSPR absorption band, as was observed under condition 2, is expected in the UV/Vis absorption spectrum of monodisperse, spherical AgNPs.

**Figure 6:**
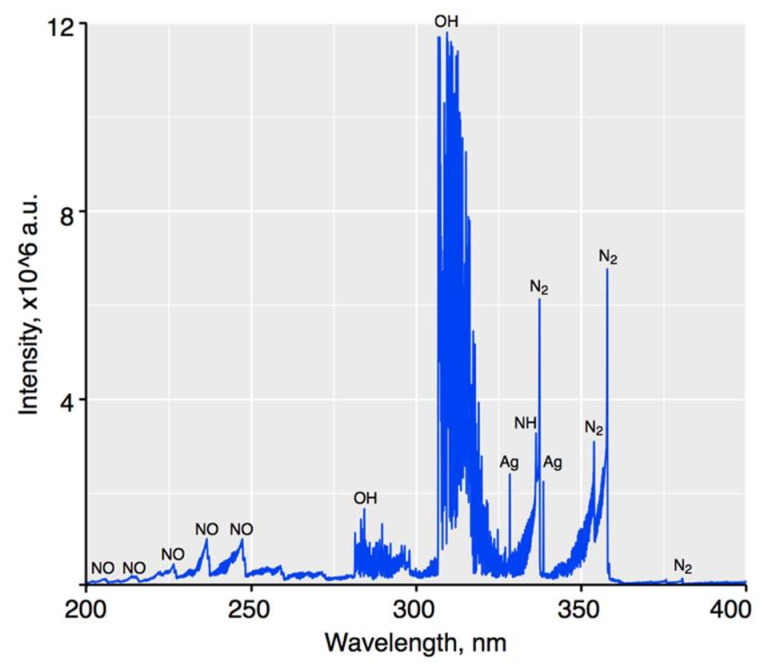
The optical emission spectrometry (OES) spectrum of pulse-modulated radio-frequency atmospheric-pressure glow discharge (pm-rf-APGD) generated under optimal operating conditions in the spectral range from 200 to 400 nm.

To finally prove that different reactive species are formed in the gas phase of the pm-rf-APGD (as a result of alternating bombardments of the surface of the flowing liquid electrode with electrons and positive ions) and may contribute to the reduction of Ag(I) to AgNPs in the liquid phase as well as the formation of other species according to the reactions presented in the Section 3.2., OES was used to acquire the emission spectra of this unique discharge system operating under condition 2. This spectrum is provided in Figure 6 and was recorded in the spectral range from 200 to 400 nm, in the near-liquid electrode zone. It was found that the UV region of the plasma-reaction system was dominated by the emission bands of N_2_ molecules (290–380 nm), which belong to the numerous transitions of the second positive system (C^3^Π_u_-B^3^Π_g_). Furthermore, bands of the NO γ-system (A^2^Σ^+^-X^2^Π) were observed in the 200–280 nm UV region. In addition, strong bands of OH molecules at 282.9 nm (the transition 1–1) and at 308.9 nm (the transition 0–0), which belong to the A^2^Σ^+^-X^2^Π system, were excited. NH molecules, belonging to the A^3^Π-X^3^Σ system, were also present in the spectrum, as determined by the presence of the band heads at 336.0 (the transition 0–0) and at 337.0 (the transition 1–1). Moreover, strong atomic Ag lines at 328.07 and 338.29 nm were identified, likely as a result of the formation of AgNPs in the liquid–plasma interfacial zone [46]. The NO and NH molecules were presumably produced as a result of the reaction of active nitrogen (N_2_ and N) with O and H radicals, respectively [47]. On the other hand, the main source of the OH radicals observed in the emission spectrum were likely dissociative processes of water (H_2_O) and its ions (e.g., H_2_O^+^ and H_3_O^+^), as was figured out on the basis of [37,38]. All reactive species, that is, NO, N_2_, OH, O, NH, N_2_^+^, and H, identified in the gas phase of pm-rf-APGD may certainly give rise to the formation of important active species in the liquid phase of the discharge that can be directly involved in the reduction of the Ag(I) ions to AgNPs.

### 3.4. Characterization of Synthesized AgNPs under Optimal Operating Conditions

An aliquot of the AgNPs synthesized under condition 2 were purified by dialysis, and the purified and non-purified AgNPs were characterized by dynamic light scattering (DLS) to determine their average size by number, polydispersity index, and ξ-potential. The average sizes by number of the AgNPs were 32.78 ± 17.55 and 41.62 ± 12.08 nm, for the purified and non-purified AgNPs, respectively (Figure 6A,B). The similarity in sizes suggested that the purification process did not significantly influence the AgNPs population.

The polydispersities of the purified and non-purified AgNPs were determined to be 0.393 ± 0.093 and 0.490 ± 0.050, respectively. The polydispersity index [48] is a measure of the width of the size distribution. When the polydispersity index value of a colloidal suspension is higher than 0.1, the dispersion exhibits polydispersity [49]. A polydispersity index value below 0.3 is favored for pharmaceutical AgNPs. Thus, the AgNPs synthesized by pm-rf-APGD exhibited moderate polydispersity. The higher polydispersity value of the non-purified AgNPs was associated with the presence of pectin, which can impact the Brownian motions and result in a higher polydispersity value being detected. The ξ-potential of the colloidal solutions was estimated in order to predict their stability, as it has been suggested that nanoparticles with negative or positive surface charges can overcome the van der Waals forces and avoid aggregation. The ξ-potentials of the purified and non-purified AgNPs were −37.8 ± 1.85 and −43.11 ± 0.96 mV, respectively. These results are supportive of these AgNPs being highly stable, consistent with the optical results reported above. Additionally, it is significant that the AgNPs were negatively charged, as this can increase their ability to access tumor cells after injection into the circulatory system [50].

**Figure 7:**
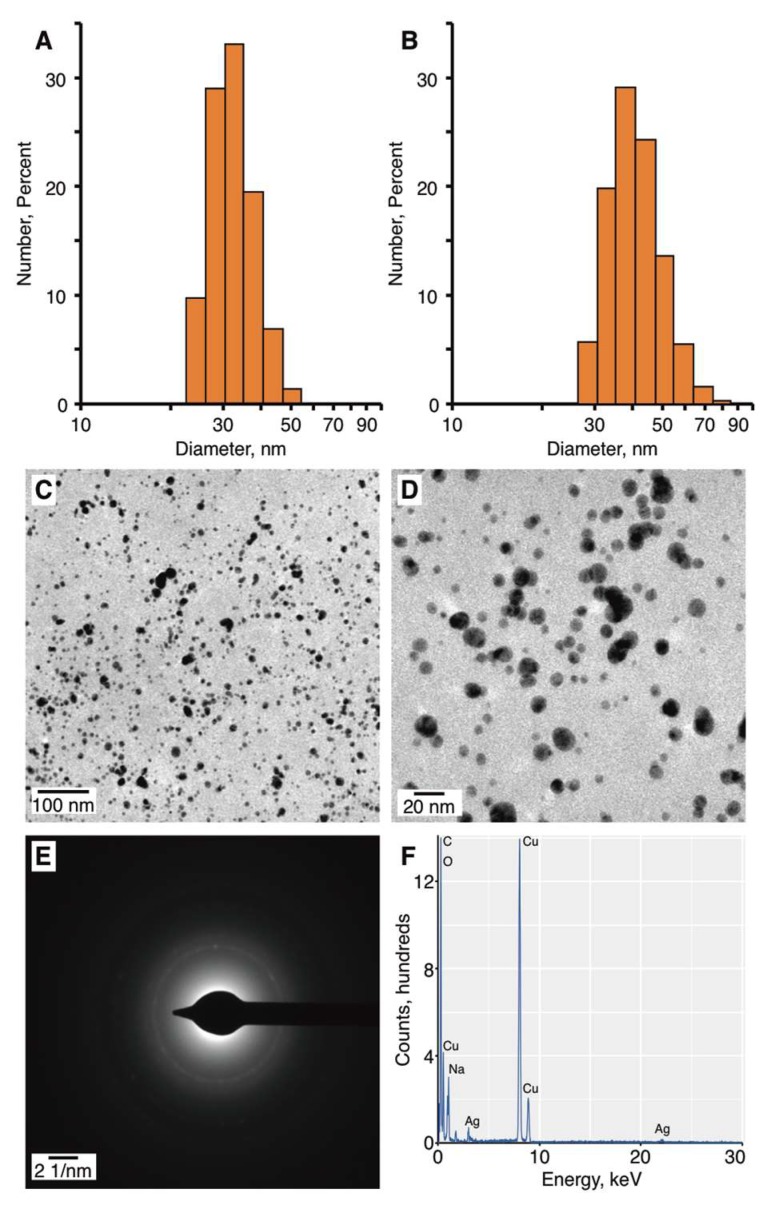
The morphology of silver nanoparticles (AgNPs) produced under optimal conditions. The histograms of size distribution by number of (**A**) purified AgNPs, and (**B**) non-purified AgNPs. (**C**,**D**) Representative transmission electron microscopy (TEM) micrographs of non-purified AgNPs, (**E**) the selected-area electron diffraction (SAED) pattern, and (**F**) the energy-dispersive X-ray spectroscopy (EDS) spectrum with prominent peaks labeled.

The size and morphology of the non-purified AgNPs were further characterized using TEM. It was observed that the AgNPs produced using condition 2 were approximately spherical in shape and non-aggregated (Figure 7C,D). On the basis of the TEM measurements (Figure 7C,D), the average size of the AgNPs was 10.38 ± 4.56 nm. It was noted that on the basis of the TEM measurements, the AgNPs appeared to be uniform in size. The differences in the sizes of the AgNPs as measured by DLS and TEM were expected and could be explained by the principals of these techniques [51]. In the case of DLS, the average size is based on the metallic core of the nanoparticles as well as on any compounds connected to the core, such as a stabilizer [51], which in this case was pectin. In contrast, size estimation as performed by TEM only considers the metallic structures [51].

SAED analysis was performed in order to determine whether the structure of the synthesized AgNPs was crystalline or amorphous. On the basis of the SAED pattern (Figure 7E), the following d-spacings were calculated: 2.334, 2.083, 1.452, and 1.350 Å. These d-spacing values correspond to (110), (200), (220), and (311) Miller indices [52]. Thus, it was determined that the synthesized AgNPs had a face-centered-cubic (fcc) crystalline structure.

EDS analysis was conducted to reveal the elemental composition of the synthesized Ag nanostructures (Figure 7F). Peaks corresponding to metallic Ag were detected in the EDS spectrum, confirming the formation of AgNPs. The presence of O and C was also detected, presumably because of the presence of pectin, an organic compound that was included in the reaction mixture. The peaks located at 8000 and 8900 keV were associated with the Cu sample grid onto which the sample was coated.

### 3.5. Influence of the Synthesized AgNPs on Necrosis of Cancer Cells

According to the American Cancer Society, cancer of the skin is the most common type of all cancers [53]. Melanoma skin cancer is responsible for the large majority of deaths related to skin cancer, with approximately 10,000 people expected to die from melanoma every year in the United States [53]. One of the most common treatments of melanoma cancer involves cell transfer therapies, based on antitumor lymphocytes and cytotoxic agents [54,55]. The cytotoxic agents might cause, for example, anemia and generation of cellular resistance [55]. A remedy to these drawbacks may be the use of AgNPs in the treatment of cancer. Previous studies have shown that nanoparticles in the range of 10–100 nm in size can have anticancer properties [56]. It is true that in vivo there are different types of cells within a tumor mass; however, all of them are activated and proliferated under a low O_2_ level as well as acidic pH conditions (hypoxic conditions and tumor microenvironment) [14,15]. Certainly, it is critical to incorporate the microenvironmental characteristics into the development of physiologically relevant in vitro models, which could provide reliable, quick, and low-cost methods for toxicity studies of the synthesized nanoparticles. The use of cell lines in the testing of the cytotoxicity of AgNPs is a common practice in the field. Therefore, the activity of the AgNPs synthesized in this study using condition 2 against the human melanoma tumor cell line Hs 294T was tested (Table 2).

**Figure 8:**
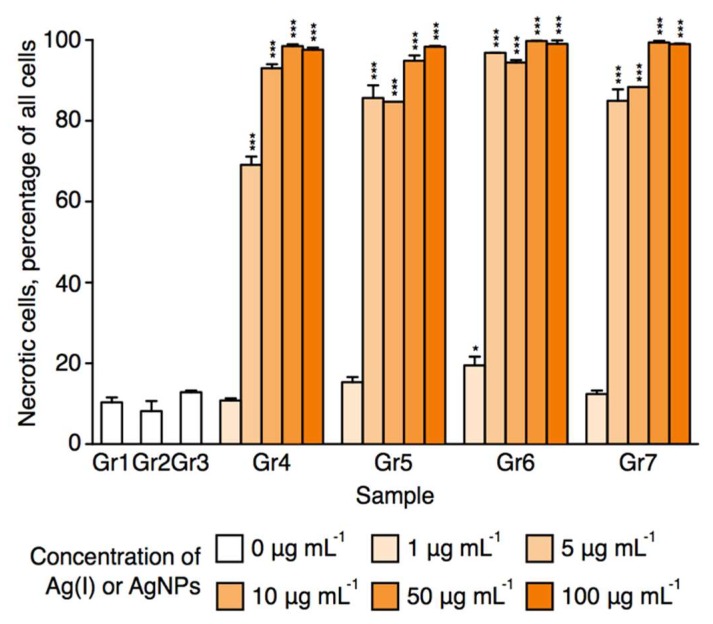
Percentage of necrotic human melanoma Hs 294T cells. Human melanoma (Hs 294T) cancer cells were treated with 1, 5, 10, 50, and 100 μg·mL^−1^ silver nanoparticles (AgNPs) or Ag(I) ions for 24 h or were treated with culture medium as a control (see Table 2 for a description of the groups). Percentages of necrotic cells were calculated as means ± the standard error of the mean of three independent experiments, each performed with triplicate wells for each treatment group. * *p* < 0.05; *** *p* < 0.0001.

As shown in Figure 8, necrosis of 70–100% of the tumor cell populations was detected following treatment with 5, 10, 50, and 100 μg·mL^−1^ purified or non-purified AgNPs compared to the control cell population treated only with culture medium (p < 0.0001). The majority of the tumor cells underwent necrosis within 24 h, with the first symptoms of tumor cell death were observable as early as 6 h after the AgNPs’ application (data not shown). No necrosis was detected when a concentration of 1 μg·mL^−1^ of purified or non-purified AgNPs was used (Figure 8). Similar results were observed when the tumor cells were treated with free Ag(I) ions as when treated with AgNPs (Figure 8). In contrast, treatment of the tumor cells with pectin alone or with pm-rf-APGD-treated water alone had no effect on the rate of necrosis compared to the control sample, which had a rate of necrosis of ~10% of the cell population (Figure 8). It was reported that AgNPs had a higher inhibition efficacy in tumor lines than in normal lines, which is due to the higher endocytic activity of tumor cells as compared to normal cells [14]. Therefore, additional experiments were performed, in which the antitumor activity of AgNPs against human fibroblast isolated from skin (MSU-1.1) as well as human endothelial cells isolated from skin (HSkMEC.2) was tested in three different concentrations of AgNPs, that is, 0.01, 1, and 5 μg·mL^−1^. No cytotoxic effect of AgNPs even for the highest concentration of 5 μg·mL^−1^ toward any normal cells of skin origin was observed.

The IC_50_ (inhibitory concentration, the concentration at which half of cells are necrotic) values were 7.8, 6.8, 4.3, and 6.6 μg·mL^−1^ for Gr4–Gr7, respectivly. This suggests that the melanoma cells were more sensitive to the solutions of Ag(I) ions before pm-rf-APGD treatment (Gr6) than to the solutions containing AgNPs. Overall, these data were consistent with the AgNPs synthesized by pm-rf-APGD inducing necrosis in human melanoma cancer cells, suggesting they may serve as an effective therapeutic agent for cancer treatment. The loss of cell viability following the AgNP treatment is believed to have been mediated through the release of Ag(I) ions within the tumor cells [57]. It is therefore not particularly surprising that treatment of the tumor cells with either AgNPs or free Ag(I) ions led to a strong reduction in cell viability, a result observed by both us and others [58]. The advantage of AgNP application lies in their selectivity toward tumor cells [59]. Whereas Ag(I) ions can display high toxicity to both cancerous and healthy cells, AgNPs can show greater specificity toward tumor cells, as the more acidic nature of their cytoplasm results in an elevated rate of Ag(I) ion release from the AgNPs relative to healthy cells [58,59]. It is important to consider that to fully determine the biological activity of nanoparticles, the correlation between material surface properties and cell functions must also be taken into account to eliminate undesired toxic effects of the nanoparticles [60,61,62,63].

## 4. Conclusions

In summary, we have developed a simple, rapid, and versatile pm-rf-APGD-based method for the size-defined synthesis of stable-in-time AgNPs. As a result of the continuous characteristic of the investigated plasma-reaction system, as well as the low-cost of its utilization, this method provides an appealing alternative to those that are presently applied for the production of high amounts of long-term stable Ag nanostructures. We believe further development of this plasma-reaction system could lead to a high-volume AgNPs-synthesis method. The possibility of controlling the properties of AgNPs should allow for further applications, not only in the necrosis of human melanoma cancer cell lines, but also, for example, in the inactivation of pathogenic bacteria.

## Figures and Tables

**Table 1:** The Box–Behnken response surface design with actual and coded values of operating parameters of the pulse-modulated radio-frequency atmospheric-pressure glow discharge (pm-rf-APGD) plasma-reaction system for synthesis of silver nanoparticles (AgNPs), along with the randomized run order and the response, i.e., *λ*_max_ of the localized surface plasmon resonance (LSPR) absorption band of AgNPs: (A) the flow rate of the flowing liquid electrode solution, (B) the precursor concentration, (C) the pectin concentration, (D) the frequency of pulse modulation of rf current, and (E) the duty cycle.

Run Order	Actual (Coded) Levels of Operating Parameters					Response *λ*_max_ (nm)
	A (mL·min^−1^)	B (mg·L^−1^)	C (%(*m*/*v*))	D (Hz)	E (%)	
1	6.0 (+1)	350 (0)	0.25 (0)	1500 (+1)	50 (0)	401.9
2	4.5 (0)	350 (0)	0.25 (0)	500 (−1)	70 (+1)	404.0
3	3.0 (−1)	350 (0)	0.25 (0)	1500 (+1)	50 (0)	404.0
4	3.0 (−1)	350 (0)	0.25 (0)	500 (−1)	50 (0)	404.0
5	6.0 (+1)	350 (0)	0.00 (−1)	1000 (0)	50 (0)	406.1
6 ^a^	4.5 (0)	350 (0)	0.25 (0)	1000 (0)	50 (0)	405.1
7	4.5 (0)	500 (+1)	0.00 (−1)	1000 (0)	50 (0)	402.5
8	4.5 (0)	200 (−1)	0.25 (0)	1500 (+1)	50 (0)	400.3
9	4.5 (0)	500 (+1)	0.25 (0)	500 (−1)	50 (0)	415.4
10	4.5 (0)	500 (+1)	0.50 (+1)	1000 (0)	50 (0)	411.8
11	4.5 (0)	350 (0)	0.50 (+1)	1500 (+1)	50 (0)	399.7
12	4.5 (0)	200 (−1)	0.25 (0)	500 (−1)	50 (0)	397.2
13	4.5 (0)	350 (0)	0.50 (+1)	1000 (0)	30 (−1)	394.7
14	4.5 (0)	350 (0)	0.00 (−1)	1000 (0)	30 (−1)	399.3
15	3.0 (−1)	350 (0)	0.50 (+1)	1000 (0)	50 (0)	409.0
16	6.0 (+1)	350 (0)	0.50 (+1)	1000 (0)	50 (0)	394.2
17	4.5 (0)	350 (0)	0.00 (−1)	1500 (+1)	50 (0)	400.8
18	3.0 (−1)	500 (+1)	0.25 (0)	1000 (0)	50 (0)	412.4
19	3.0 (−1)	350 (0)	0.25 (0)	1000 (0)	30 (−1)	404.0
20	4.5 (0)	350 (0)	0.50 (+1)	500 (−1)	50 (0)	401.0
21	4.5 (0)	350 (0)	0.00 (−1)	1000 (0)	70 (+1)	406.3
22	4.5 (0)	500 (+1)	0.25 (0)	1000 (0)	70 (+1)	415.9
23	6.0 (+1)	350 (0)	0.25 (0)	1000 (0)	70 (+1)	397.0
24	3.0 (−1)	350 (0)	0.25 (0)	1000 (0)	70 (+1)	403.1
25	4.5 (0)	200 (−1)	0.50 (+1)	1000 (0)	50 (0)	421.5
26	6.0 (+1)	350 (0)	0.25 (0)	500 (−1)	50 (0)	394.6
27	4.5 (0)	350 (0)	0.00 (−1)	500 (−1)	50 (0)	415.1
28	6.0 (+1)	500 (+1)	0.25 (0)	1000 (0)	50 (0)	411.4
29	4.5 (0)	350 (0)	0.25 (0)	1500 (+1)	30 (−1)	426.2
30	4.5 (0)	500 (+1)	0.25 (0)	1000 (0)	30 (−1)	407.7
31	6.0 (+1)	350 (0)	0.25 (0)	1000 (0)	30 (−1)	405.9
32	4.5 (0)	200 (−1)	0.25 (0)	1000 (0)	30 (−1)	384.3
33	3.0 (−1)	350 (0)	0.00 (−1)	1000 (0)	50 (0)	419.8
34 ^a^	4.5 (0)	350 (0)	0.25 (0)	1000 (0)	50 (0)	397.5
35	4.5 (0)	350 (0)	0.25 (0)	500 (−1)	30 (−1)	392.5
36	4.5 (0)	200 (−1)	0.25 (0)	1000 (0)	70 (+1)	390.1
37	6.0 (+1)	200 (−1)	0.25 (0)	1000 (0)	50 (−1)	399.7
38 ^a^	4.5 (0)	350 (0)	0.25 (0)	1000 (0)	50 (0)	395.3
39	4.5 (0)	350 (0)	0.50 (+1)	1000 (0)	70 (+1)	403.1
40	4.5 (0)	200 (−1)	0.00 (−1)	1000 (0)	50 (0)	403.5
41	4.5 (0)	500 (+1)	0.25 (0)	1500 (+1)	50 (0)	411.0
42	4.5 (0)	350 (0)	0.25 (0)	1500 (+1)	70 (+1)	406.2
43	3.0 (−1)	200 (−1)	0.25 (0)	1000 (0)	50 (0)	410.6

^a^ Center point.

**Table 2:** Experimental groups used in the in vitro tests.

Serial No.	Treatment/Compound
Group 1 (Gr1)	Cells treated with medium alone
Group 2 (Gr2)	Cells incubated with pectin at a concentration of 2500 mg·L^−1^
Group 3 (Gr3)	Cells incubated with pulse-modulated radio-frequency atmospheric-pressure glow discharge (pm-rf-APGD) activated water
Group 4 (Gr4)	Purified silver nanoparticles (AgNPs) solution
Group 5 (Gr5)	Solutions of Ag(I) ions and pectin before pm-rf-APGD treatment
Group 6 (Gr6)	Solutions of Ag(I) ions before pm-rf-APGD treatment
Group 7 (Gr7)	Non-purified AgNPs solution, containing unreacted Ag(I) ions

**Table 3:** Outputs of analysis of variance (ANOVA) and the lack-of-fit test for the response surface regression model established using the forward-selection-of-terms algorithm (α to enter = 0.25) for the pulse-modulated radio-frequency atmospheric-pressure glow discharge (pm-rf-APGD) plasma-reaction system used for synthesis of silver nanoparticles (AgNPs) ^a^.

Source of Data	*DF*	Adjusted *SS*	Adjusted *MS*	*F*-Value ^b^	*p*-Value
Model	6	988.09	164.68	2.84	0.023
Linear	4	657.69	164.42	2.84	0.038
*A*	1	196.70	196.70	3.39	0.074
*B*	1	410.06	410.06	7.08	0.012
*D*	1	43.23	43.23	0.75	0.393
*E*	1	7.70	7.70	0.13	0.718
Square	1	82.33	82.33	1.42	0.241
*E*^2^	1	82.33	82.33	1.42	0.241
Two-way interactions	1	248.06	248.06	4.28	0.046
*DE*	1	248.06	248.06	4.28	0.046
Error	36	2086.15	57.95	-	-
Lack-of-fit	34	2033.27	59.80	2.26	0.354
Pure error	2	52.88	26.44	-	-
Total	42	3074.24	-	-	-

^a^ *DF*: Degrees of freedom. *SS*: Sum of squares. *MS*: Mean of squares. *A*: The flow rate of the flowing liquid electrode solution. *B*: The precursor concentration. *D*: The frequency of pulse modulation of radio-frequency (rf) current. *E*: The duty cycle. ^b^ The value of the F-test for comparing model variance with residual (error) variance.
